# Journeys: understanding access, affordability and disruptions to cancer care in India

**DOI:** 10.3332/ecancer.2022.1342

**Published:** 2022-01-13

**Authors:** Soumitra Shankar Datta, Soumita Ghose, Manisha Ghosh, Amruta Jain, Sumedha Mandal, Sayan Chakraborty, Carlo Caduff

**Affiliations:** 1 Department of Palliative Care & Psycho-oncology, Tata Medical Center, Major Arterial Road, New Town, Rajarhat, Kolkata 700160, India; 2 MRC Clinical Trials Unit, Institute of Clinical Trials and Methodology, University College London, 90 High Holborn, London WC1V 6LJ, UK; 3 Department of Administration and Policy, Tata Medical Center, Major Arterial Road, New Town, Rajarhat, Kolkata 700160, India; 4 Department of Global Health & Social Medicine, King’s College London, Bush House, 40 Aldwych, London WC2B 4BG, UK; a https://orcid.org/0000-0003-1674-5093; b https://orcid.org/0000-0003-0084-1283; c https://orcid.org/0000-0003-4505-3408; Photobook on the patient journey: https://ecancer.org/en/news/21422-journeys-a-photo-essay

**Keywords:** cancer care, oncology, LMIC, disruptions, affordability, access

## Abstract

**Background:**

Much of the global cancer burden is in low- and middle-income countries (LMICs). Along with the high incidence of cancer, most LMICs have unevenly distributed health care resources. This study is a qualitative exploration of the journey of patients accessing cancer care in India and their caregivers.

**Methods:**

The study followed a cross-sectional qualitative design. Participants were recruited by stratified purposive sampling, and all common cancers in India as reported by the GLOBOCAN database were included in the study. Consenting patients and their caregivers were interviewed using in-depth interview techniques. The data was analysed using principles of qualitative content analysis.

**Results:**

Cancer patients (*n* = 100) and their caregivers (*n* = 48) were interviewed for the study. The six themes that emerged were related to a) the journey of patients to access care, b) the psychological journey of patients, c) stigma of cancer patients, d) decision-making and adherence to treatment, e) economic costs of cancer care and its impact and f) modifiers to accessing cancer care.

**Conclusions:**

Planning and policymaking in the future of cancer care delivery need to consider the views expressed by the cancer patients and their caregivers as regards to access, adherence and disruptions to cancer care in India. Future policies will hopefully address some of the difficulties faced by patients.

## Background

In 2020, the total number of new cancer patients in India was 1,324,413, and the number of cancer patients who died in the same year in India was 851,678 [1]. Globally a large proportion of cancer-related mortality is from low- and middle-income countries (LMICs) including India [2, 3]. Affordability and access to cancer care remain deficient especially for the poor [4]. Even when free-of-cost cancer screening is available, the acceptance remains low in the high-risk groups [5]. Social barriers to early detection of women’s cancer include low female empowerment and misconceptions [6], fear of cancer, lack of approval from partners to access health care and absence of a supportive social milieu for women to participate in a screening programme [7]. Thus, alongside the provision of affordable and accessible health care facilities [8], there is an urgent need to develop a deeper understanding of the patient preferences, early treatment facilitators, social barriers and enablers of cancer care in India [9]. This will, hopefully, make way for an affordable health care infrastructure.

Oncology is increasingly envisaged to be person-centred, and encourages patients to actively participate in decision-making [10, 11] and remain engaged in managing the disease and its symptoms [12]. A global review, addressing cultural aspects of cancer care, suggested that clinicians and policymakers need to understand the personal values and beliefs systems of patients while designing and offering oncology services [13]. Family of the patient also play a key role in cancer care in India [14]. Skills to understand patient preferences, and making the care person-centred, have been identified as a crucial training gap in oncology [15]. Oncology clinicians benefit from developing cultural competencies in making oncology care individualised to the needs of a specific patient [16] and at the same time understanding the role of the family members of the patient who are often the primary sources of support for the patient [14]. Alongside disease control, the health systems should be adept at addressing the social, emotional and financial impact of cancer care [16].

The Global Burden of Diseases data has highlighted the inter-regional variations for various cancer types in India [17]. Access to oncology services is limited in rural as well as some urban areas in India resulting in patients travelling long distances to visit cancer centres mostly located in cities [8, 18]. Patients often visit several different hospitals before reaching a cancer centre [19].

Of the several research studies on cancer services in LMICs [20], very few studies have focused on the aspects of cancer care from the point of view of the patient [21, 22]. This qualitative explorative study focuses on the cancer journey of the patient and the family. The three broad areas that we explored were 1) how patients and their caregivers decided about what they needed to do once the patient had symptoms, 2) how was their journey through the disease and treatment and 3) what was the impact of cancer on their broader trajectories of life.

## Methods

### Study overview and design

This cross-sectional hospital-based qualitative study was conducted to generate robust empirical evidence of cancer care provisions in India from the perspective of patients and caregivers for all common cancer types for men and women in India. Semi-structured interview guides were developed for patients and caregivers. The interviews broadly covered how patients and their caregivers decided about access to care, how was their journey through the disease and treatment (including their need to be constantly mobile due to the need to access cancer care) and what was the overall impact of cancer on their lives. The study also attempted to identify the treatment-seeking pathways of cancer patients and explore the reason(s) for delays and roadblocks in seeking timely and effective treatment.

The interviews also covered the patient’s perspectives about the healthcare facilities that they have visited, their views on the quality of care received, their perception related to direct and indirect costs of cancer care and the overall impact of cancer on the broader life of the patient. The qualitative content analysis (QCA) approach was used. The study was approved by the Institutional Ethics Committee of Tata Medical Center, Kolkata (TMC/EC/18/17).

### Study setting

The present study was conducted in Tata Medical Center, Kolkata, which is a comprehensive tertiary cancer care Institute, having all subspecialities of oncology, catering to patients from a geographically wide catchment area in eastern India and neighbouring countries of Bangladesh, Nepal and Bhutan. The hospital is a registered charity and has provisions for subsidised treatment facilities for a proportion of patients. Patients from economically deprived backgrounds can access funds to cover their treatment costs following a structured assessment by a dedicated social services department. In 2019, the hospital had seen more than 20,000 new cancer patients and during the pandemic of SARS-CoV-2 (Severe acute respiratory syndrome 2) in 2020, the hospital functioned without any major interruptions.

### Researchers

The research team consisted of a consultant psychiatrist, a public health expert, an academic anthropologist, a psychologist and a social worker. Two of the senior researchers had considerable expertise in conducting and supervising qualitative research. The two research interviewers (a psychologist and a social worker with experience of working in the field of oncology) were trained in the methods of qualitative in-depth interviewing techniques. Ongoing research supervision was provided by the senior researchers. None of the researchers who were part of the study were part of the treatment team managing the patient and this provided a degree of neutrality and opportunity for participants to open up during the interviews.

### Selection of participants

The study followed a stratified purposeful sampling strategy. Patients with all common cancer types as reported by the World Health Organization in the Global Cancer Observatory (GLOBOCAN) 2020 data for India [1] were included. The recruitment of study participants happened between September 2017 and December 2017. During this period, the hospital had seen 5,628 new cancer patients. The participants were selected using purposive sampling from outpatient clinics run by various sub-specialities of oncology. The proportional recruitment of patients was stratified as per proportional numbers of patients of each cancer type reported by GLOBOCAN data and also on the principles of data saturation. Effort was taken to include a diverse range of patients and caregivers of both genders and patients from various stages of the treatment by maintaining a log of recruitments. Recruitment of a particular stratum was stopped after achieving data saturation. Sample size was thus determined by the principles of data saturation. Some patients were newly diagnosed with cancer, others were undergoing treatment for cancer and some others had already completed treatment and were under follow-up. All participants were interviewed face to face by the trained researchers of the study team.

**Inclusion criteria:** Adult patients with cancer confirmed by a histopathological diagnosis and their family members who were fluent in Bengali, Hindi or English, which are the common languages spoken by patients in Eastern India, were eligible to participate.

**Exclusion criteria:** Those patients who were deemed to be physically too unwell by their treating team, to participate in a qualitative interview, were not eligible to participate in this study.

### Recruitment to the study

Participation in the study was voluntary. Access to patients and family members was through the clinicians treating the patients in out-patient clinics of Tata Medical Center, Kolkata. Doctors working in the out-patient clinics introduced the patients to the researchers. The researchers explained the study to the patients, provided a written information leaflet in the vernacular of the patient and recruited the participants to the study after obtaining written informed consent.

### Qualitative interviews

All interviews were conducted in a secure and quiet location away from the busy clinics to provide privacy to the patients. There were no outsiders except the researcher and the participant present during the interview. No member of the treatment team was present during the interview. The interviewers asked non-intrusive, open-ended questions and did not interrupt the narratives provided by the participants. This gave the participants as much control as possible, over what they wanted to talk about and when to shift the topic of conversation. The patients and relatives were probed on their experience of engaging with health services, how they decided on accessing health care and how did they prioritise competing needs. Issues raised included their preference for travelling from home to the cancer centre and impacts of cancer on the patient and their family member. The interviewers had access to the interview guide that acted as an aide-memoire to help the interview process. Following the initial interviews, additional questions were added to the interview guide to explore new but related topics in line with the qualitative research methods. All interviews were conducted between September 2017 and December 2017. The interviews of patients and caregivers lasted on an average 44 minutes ± 12 minutes. All interviews were audio-recorded and transcribed. The data included a) qualitative in-depth interviews of patients and b) brief socio-demographic data of the participants to supplement and contextualise the qualitative data.

### Data analysis

The study was conducted with the methodological orientation of content analysis. QCA has been used in a wide range of disciplines and was felt particularly suitable for the large volume of data for the present study. QCA aims to generate meaning of the material analysed [23] and has been found to be suitable for studying sensitive phenomenon and when conducting exploratory work on a topic [24]. The qualitative audio-recorded interviews were transcribed verbatim by MG and AJ and the transcripts were anonymised. Data analysis was done manually without using any specific software. The two researchers who coded the data were experienced qualitative researchers with multiple publications using the qualitative methodology. The steps of building a coding frame as suggested by Schreier [23, 25] were followed, namely, a) selecting, structuring and generating categories; b) defining categories; c) revising and expanding the frame. The steps of revising and expanding the frame are useful as it is seldom possible to build the final coding frame at the beginning. Following this methodology, a coding frame was built by SG and SSD. Subsequently, the two coders (SG and SSD) independently divided the interview material into the coding frame that went through a few iterations of modifications. After the coding of the first 30 interviews and constructing the coding frame, the validity of the hypothesis generated was checked by the generation of similar themes emerging from later interviews and the coding frame was further refined. Knowing that cancer patients may often face unforeseen medical emergencies, the study team did not get back to the participants after transcription of the interviews or after analysis. This was followed by further interpretation of the data by SG and SSD adapting an ‘empathic’ stand [26] and in the process to remain as close to the data as possible.

### Results

The study included 148 participants comprising 100 patients with cancer and 48 caregivers of cancer patients. Data collection continued for patients and care givers as per the criteria of meeting data saturation as is conventional in qualitative research. The mean age of patients and caregivers recruited for the study was 61.3 years (range: 25–89) and 44.2 years (range: 21–75), respectively. As evident from Table 1, younger patients were often accompanied by older caregivers as their parents or elder siblings and older patients were accompanied by their younger caregivers as their children. Family members (husband, wife, sons and daughters) constituted 69% (33/48) of the caregivers interviewed. Around half (48%) of the patients had graduate-level qualifications and a quarter (25%) had no formal education and thus the sample of patients represented a wide spectrum in their respective socio-economic and educational backgrounds. Men constituted 35% (35/100) of the patients recruited to the study and 62% of care givers (30/48). Thirteen percent of the patients (20/148, 13.51%) recruited for the study had financial support from government or private donor funds to support the cost of cancer treatment. All the common cancers in India (breast cancer, head and neck cancers, gynaecological cancers, lung cancer and others) were represented in this study (Table 1). Proportional number of various types of cancer patients was recruited based on the approximate proportion of cancer patients reported by GLOBOCAN [1].

**Figure 1.**
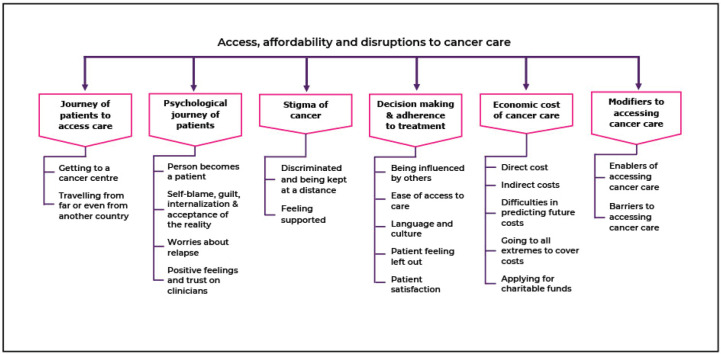
Coding tree for access, affordability and disruptions to cancer care.

Six broad themes and 20 subthemes emerged from the qualitative analysis of the data. The broad themes were on the a) journey of patients to access care, b) psychological journey of patients, c) stigma experienced by some cancer patients, d) decision-making and adherence to treatment, e) economic costs of cancer care and f) modifiers to accessing cancer care. The coding tree of the interviews is represented schematically in Figure 1.

### Theme 1: Journey of patients to access care

#### Getting to a cancer centre

After the onset of symptoms, most patients had approached a local physician near home. Often, they had been reviewed by more than one doctor before reaching a cancer centre. However, for some patients, this process was relatively quick, whereas for others the journey was fraught with difficulties.

***‘She (the patient) noticed that her nipple got pushed inside. Because we don’t have any lady in our home, she did not tell anyone at first. She showed it to a neighbor, who said that this needs to be reviewed by a doctor immediately. Then we went to a local doctor. The local doctor said we must go to Kolkata. Then we first went to XXXX hospital who suggested that patient needs surgery, but this may not be successful. As we could not trust this judgment, and that is why we came to XXXX in the same month’ (Caregiver #6, Caregiver of breast cancer patient).***

Patients often had a complicated road towards their diagnosis of cancer by visiting multiple centres, having to see many doctors for a confirmed diagnosis. Some patients underwent inadequate and incomplete treatment. Appropriate management of the disease was often delayed. Eventually, patients reached a cancer centre where at times the patient had to undergo another completion surgery thus being burdened with two surgeries rather than just one, adding to the costs.

***‘We had enquired if I needed to undertake FNAC. The doctor had reassured us that I should not have any reasons to worry. Subsequently, he operated on me, and following that the biopsy results (histopathology following surgery) diagnosed me to have cancer. Then my father brought me to XXXX to see Dr XX. After I saw Dr. XX she examined me and said that my glands in my axilla were quite swollen and my earlier doctor had not taken them out. She would like to operate and take them out. Subsequently, I had my (second) surgery’ (Patient #2, Breast cancer patient).***

Inadequate diagnosis, insufficient explanation, lack of communication, lack of coordination and incoherent referral pathways often contributed to the confusions experienced by the patients and their families.

***‘…. scan report detected 2 cysts but the first FNAC report was wrong. …. So the doctor told us to do the FNAC again...So we were really confused. All the doctors were saying different things. The test reports were also wrong. Then we went to XXX. There they performed a lot of tests on her. And the doctor said it looks like cancer. So we came back to Calcutta and went to a doctor in XXXX, ….. He said you have to redo the Core Biopsy….’ (Patient #24, Husband of lung cancer patient)***

One patient mentioned that he did not understand seriousness of his condition when he initially presented to the health system. He seemed to have been scared, desperate to access the best possible care by travelling between cities but unsure how to do that.

***‘Almost a year and a half ago, suddenly I fainted while walking on the road. I had frothing from my mouth, my arms and legs became stiff, and I lost consciousness. They took me to a local hospital and they advised for CT scan. That night I took an 11:30 flight and came back to Calcutta and after coming back I visited a physician. He insisted that I should consult a neurosurgeon, but I just carried on with my life’ (Patient #78, renal cancer patient).***

Some patients considered getting treated at the government-supported primary healthcare system but eventually reached specialised cancer centre. There were both positive and negative views when patients compared government and private health care setups. In India, both these systems run in parallel.

***‘The govt medical college was good too but we still came here because everyone knows this as a good cancer hospital. Getting well was my priority. My family wanted better treatment’ (Patient #25, Hodgkin’s Lymphoma patient).***

***‘Because there are so many patients in government hospitals your treatment is always delayed. That’s why people are scared to keep their patients in government hospitals’ (Caregiver #108, stomach cancer patient).***

One patient was referred to a specialist cancer centre after relapse was diagnosed.

***‘After the relapse, the doctor in xxxx said he can’t treat anymore, because this is beyond his expertise. He then referred me to XXXX. I could not understand why he decided to treat me in the first place’ (Patient #113, Lymphoma patient).***

#### Travelling from far or even from another country

Patients also came to India for cancer treatment from neighbouring countries such as Bangladesh. One such patient mentioned the cost of treatment to be the main reason for accessing treatment from India. The treatment would have costed more in their home country.

***‘……The cost of medication is very high in Bangladesh. If I had bought the same medications in Bangladesh it would have cost me almost three times as much. Altogether the cost (of treatment) in Dhaka is much higher compared to the cost here. So if I do the costing my coming here made sense….’ (Patient #16, endometrial cancer patient)***

### Theme 2: Psychological journey of the patients

#### A ‘person’ becomes a ‘patient’

More than the fear of the disease, patients expressed shock as they came to know of their disease. Many of the patients expressed disappointment that despite leading a very healthy life they were diagnosed with cancer. Their search for an answer about why they got the disease made some of them goes through a spiritual crisis.

***‘I often ask God, why did I get such a disease?’ (Patient #1, breast cancer patient)***

***‘…..I used to lead a normal life. I never drank or smoked. I even avoided spicy or oily food’ (Patient #67, Pancreatic cancer patient).***

***‘I cannot imagine what I am carrying around within me’ (Patient #15, rectal cancer patient)***

#### Self-blame, guilt, internalisation of the disease and acceptance of the reality

Patients had different ways of dealing with their diagnoses. While some accepted it as mere fate, others treated it as a punishment from God. Few of them mentioned that they relied on the strength they obtained from their faith to come out of this crisis.

***‘God has given me this disease. I can do nothing about it’ (Patient #68, rectal cancer patient)***

***‘There is God. So, everything is under control’ (Caregiver #19, Caregiver breast cancer patient)***

Many patients were troubled about having to stop their normal life for this disease. In the initial part of the treatment, some patients continued in their job. Over time, once they became progressively unwell they had to stop working. One of the patients we interviewed seemed to have lost his anchor in life after he had to stop going work. He indirectly conveyed during his interview that only thing he can do now is wait until he dies.

***‘But I used to go for work within 5 days after chemo. Because of the financial problem I used to force myself to go to work. Every one used to ask me why have I come to office so soon. Actually, I have been with cancer patients from a very young age. That’s why I was never scared of disease. Many get scared of cancer, I never had that. I treated this as if it’s a simple fever. But now my health is telling me that I have ‘cancer’. I am in mental distress as I am sitting at home, eating four times a day without being able to do anything (started crying). This is a very painful situation (pause) I think death is better than this’ (Patient #72, lung cancer patient).***

Patients also felt guilty for being a source of inconvenience to their family members having to spend so much money for treatment as well as undergo other inconveniences such as missing out from work, studies, travelling far away.

***‘It’s because of my illness; my son is missing out on his exams. It’s all because of me that he is not getting a job...’ (Patient #1, breast cancer patient)***

#### Worries about relapse

Not only patients but caregivers were also worried about the disease coming back. This could be the case even if the patient was getting treated with curative intent.

***‘It is scary when you know it’s cancer. Everyone knows cancer often relapses after 5 years. But you must keep fighting’ (Caregiver #141, Caregiver of oral cancer patient).***

Other respondents talked about patients being psychologically devastated after relapse was diagnosed.

***‘At the beginning, when he got diagnosed (with cancer) although he was very sick physically, he remained mentally strong. But now after the relapse, he has broken down both mentally and physically’ (Patient #57, colon cancer patient).***

#### Positive feelings and trust on clinicians

Many of the patients were very grateful to the doctors who had treated them and for explaining the treatment to them.

***‘I trust my doctor. I respect her a lot. My cancer is just restricted to the nipple so I suggested that they should remove it completely. Who knows what will happen in the future. There was another test conducted post-surgery. Based on the reports the doctors informed that there is nothing more to worry about’ (Patient #1, breast cancer patient).***

***‘…..Now I am keeping much better under his treatment. I am generally really grateful to doctor XX. I would say this sort of case is completely dependent on Gods like Dr. XX. Those who are in touch with cancer hospital they are part of God….’ (Patient #78, renal cancer patient)***

### Theme 3: Stigma of cancer

#### Discriminated and being kept at a distance

Some patients experienced discrimination following the diagnosis of cancer. A few women with cancer reported that people stared at them and laughed at their appearance. One patient shared that the belief of cancer being caused by supernatural causes or believing that it is a transmissible infective illness contributed to the stigma.

***‘They believe that if the person has cancer, there are germs in her body. I know in reality it is not like that. But they feel disgusted. Those people don’t even sit next to you or be with you in the same room. They won’t eat with you. They may also believe that black magic caused cancer…’ (Patient #148, breast cancer patient)***

**Feeling supported**

Not everyone experienced discrimination. Some patients felt that their friends and family stood by them in their cancer journey. Several patients felt supported by the wider community.

***‘My friends helped me a lot. Not everyone could come to the hospital, but I felt supported by everyone’ (Patient 14, 59 years, female, breast cancer patient).***

***‘Nobody stigmatizes this disease. Everyone stood by me, my wife, my son, my daughter’ (Patient 110, 61 years, male, head & neck cancer patient).***

### Theme 4: Decision-making and adherence to treatment

#### Being influenced by others

Decision-making by patients regarding treatment options had to do much beyond the nature of the disease. Decision-making was influenced by the domicile and milieu of the place they lived, availability of local health facilities, the experience of the service users with health care providers and the opinion of people whom the patient trusted as advisers.

The experiences of friends, family, neighbours, extended family seemed to have played an important role in the decision-making process. The experiences of knowing others with cancer who got treated in a particular centre was one of the key factors in choosing a treatment centre.

***‘There wasn’t any referral. My uncle knew about this hospital and many people from Asansol have come here for treatment. And we knew that they offer good treatment. That’s why we didn’t go anywhere else. We directly came here’ (Caregiver #33, caregiver of breast cancer patient).***

At other times, someone in the family knowing a particular doctor also influenced the choice.

***‘My nephew is a doctor, and he has a very good relationship with Dr. X in XX hospital. So, when he called Dr. X, he immediately called me the next day and reviewed me’ (Patient #78, renal cancer patient).***

There were situations where patients relied on the opinion of others who believed in alternative methods of medicines. This led them to opt for a certain alternative treatment modality often at the cost of the cancer progressing. They ultimately reconsidered their choices later on, but this often resulted in a delay in starting treatment.

***‘People were giving all sorts of suggestions like taking homeopathic medicines. So, we went to XXXX, to get Ayurvedic medications. I took those medicines for one month, but it did not help. Only after that my father was convinced about the need for surgery’ (Patient #121, laryngeal cancer patient).***

#### Ease of access to care

Travelling long distances from home to tertiary cancer hospitals was a barrier to access appropriate cancer care. Other logistic challenges included adjusting to a new city, being away from home, lengthy commutes, staying away from home for a long period, incurring additional expenditures for travel and lodging, etc. These came out to be decisive factors regarding accessing treatment from a given centre at any specific point of time. Some patients chose a treatment facility closer to home.

***‘Presently she is not physically strong enough to travel to XXXX. If we take her far away from home for better treatment, her general and emotional health will get compromised’ (Patient #52, Caregiver of breast cancer patient).***

#### Familiarity with the language and culture of the place where the hospital is located

Patients, while making treatment choices, were influenced by factors related to their lifestyle and living preferences. Many of them preferred to go to the centre located at a place with a language and culture that they are familiar with. These could be around food habits or religious practices. The patients and their caregivers found living away from home hard enough. They did not want to compromise on these seemingly small but significant aspects of their day-to-day lives.

***‘…We would have preferred Mumbai. But we didn’t go there because of two reasons. We are very sensitive about food. Although she (patient) is undergoing chemotherapy, she is still able to cook her food as per her preferences. There are a lot of cultural differences between home and Mumbai. But the culture in Bangladesh and Calcutta is almost 80% similar’ (Caregiver #22, caregiver of ovarian cancer patient).***

#### Patient feeling left out of decision-making

Most patients immensely valued good communications by their treating medical teams and preferred to know about the details of the treatment they are undergoing. They felt let down if this did not happen. Many of the patients felt that they were not made part of the decision-making process about their illness.

***“I told them that I won’t get scared as I work in a similar profession. But they didn’t tell me anything. They (doctors) discussed amongst themselves, and said ‘go to the other room, we have uploaded all the information on the computer’” (Patient #148, breast cancer patient)***

Some patients also specifically mentioned that they would have preferred to be more involved in the care.

***“He (doctor) asked me how I am doing. I asked the doctor ‘can you please tell me what my improvements are?’ Nobody is telling me clearly what treatment I am receiving? The only thing they said at the beginning was that this disease cannot be cured and that I have a very bad disease. But does that mean the treatment will continue indefinitely and I may not get any benefit? It is difficult for me to accept this” (Patient #8, gallbladder cancer patient).***

***‘Today the doctor advised me to undergo so many tests, but he couldn’t give an answer to any of our queries. He couldn’t even say what the disease is’ (Patient #19, breast cancer patient).***

#### Patient satisfaction and trust leading to treatment engagement

Doctors who took time to explain the disease in a way that the patient understood and explained the care plan could motivate the patients to undergo treatment with a more positive mindset. This made a significant change in the patient’s attitude towards the treatment.

***‘I am getting proper treatment here. If I didn’t get proper treatment, then I would have died. Today Dr. XXXX and Dr. YYYY very carefully explained to me in detail the treatment I am receiving. I am very satisfied with that. Because I understood that they are trying to cure me. Hence, I am not thinking of going anywhere else’ (Patient #94, 70 years, male, Anal cancer patient).***

Often this feeling of trust helped in treatment adherence.

***‘Many people gave me a lot of suggestions and sometimes they were completely contradictory to each other. But I am just following what was suggested by my doctor’ (Patient #67, Pancreatic cancer patient).***

***‘We are villagers. Everyone fears cancer. Earlier we believed that if a person gets cancer, nothing much can be done. But my mother is almost cured today. I trust this hospital. My mom was in a very bad condition, but today she is almost cured’ (Caregiver #58, Caregiver of breast cancer patient).***

### Theme 5: The economic cost of cancer treatment

#### Direct cost of cancer treatment

Some of the patients and their family members had already spent a significant amount of money before being interviewed for this study. The cost ranged from $20 to as much as $26,730 in some patients. These patients were in different stages of their treatment, as well as different types of cancers while being interviewed for this study. It was interesting to see that a vast majority of patients did not have any health insurance and had to bear the cost of treatment out of pocket. Some of the patients who were early in the course of treatment were worried about the immediate and longer terms financial implications.

***‘I work in a bookstore. We have sold whatever we had in our house. Now we don’t know how we are going to bear the cost of such a big surgery’ (Caregiver #39, Caregiver of rectal cancer patient).***

Many of the patients did not have the personal financial means to organise the money for their treatment. Family members and the extended family were the biggest sources of financial support. The workplace and social circle of the patient also proved to be an effective source of funds for many. Some of the family members spontaneously spoke about the indirect costs of income loss of family members supporting the patient as well as the impact of the treatment costs on their lives.

***‘We are normal people, and we are managing somehow. For some people who are dependent on one person’s income, it’s very difficult. And this is a long-term condition. There will be regular check-ups. I need to be engaged in caring continuously for my mother’ (Caregiver #22, Caregiver of ovarian cancer patient).***

#### Indirect costs of cancer care

Patients understood that the cost of cancer treatment involved not only the direct costs but also the costs of accommodation in a new place, loss of earnings and various other costs. They said that all of this affected them.

***‘In Mumbai, we rented a room to stay. If you rent a room, it can be $6.7 or $13.4 per day. We didn’t stay in the $13.4 room. We stayed in the $6.7 one. For the 22 days that we stayed there, we paid $6.7 every day. Three of us went there and there was also the cost of food. We soon ran out of money. We were told to stay another month. The doctor said that they will give chemotherapy. We didn’t have the financial means to spend so much money. We are villagers and where will we get so much money?’ (Patient #3, ovarian cancer patient)***

One relative pointed out that loss of income for the relatives were causing significant economic hardships.

***‘My brother is also here. So, I think, time is money. As both of us are involved in the treatment, we are facing economic hardships’ (Caregiver #22, Caregiver of ovarian cancer patient).***

#### Difficulties in predicting future costs

The treatments of most of the cancers take time and may involve a series of modalities stretched sometimes over several years.

***‘Whatever we had to spend till date, we have somehow managed. But we don’t know about the future. Because that cancer journey is long. The treatment is for the long-term’ (Patient #101, thyroid cancer patient).***

***‘The doctor said around $3340 will be needed only for the surgery. Following that, we also need to bear the cost of medicines, tests, etc. We are clueless about how to manage this. Our brains aren’t working anymore…’ (Patient #68, rectal cancer patient)***

In oncology, there can be relapse or recurrence of the disease that requires further treatment. Some of the patients interviewed in this study were being treated for a recurrence. Some patients were on palliative treatment. One patient expressed his psychological distress related to the inherent uncertainty of their future treatment and its associated costs.

***‘If I need to have to cardiac bypass surgery or placement of a cardiac pacemaker, then I know the duration of my treatment and what will be the cost of it. God knows, in oncology, you might have to come back after 6 months or 1 year and if cancer comes back in some other form. The common man suffers. There is no guarantee’ (Caregiver #11, Caregiver of ovarian cancer patient).***

#### All hands-on deck: going to all extremes to cover costs

A significant number of patients interviewed in the study had already spent almost all their lives’ savings towards treating this disease. By the time they were interviewed in this study, many of them had to sell off houses, land, jewellery, cattle and any other forms of assets to meet the cost of treatment.

***‘No one helped me. I bought that piece of land after a lot of struggle but now I had to sell it…’ (Patient #111, cervical cancer patient)***

***‘Yes, we have sold the gold jewelry. Our two daughters had two necklaces. They are married now. Those 2 gold necklaces have been sold as well’ (Caregiver #89, stomach cancer patient).***

On asking one of the above caregivers if they have borrowed money from anyone, the person gave a poignant reply via a rhetorical question.

***‘No one will give him a loan. Who will loan money to someone who is fighting with death?’ (Caregiver #89, stomach cancer patient)***

There were, often, far-reaching consequences such as loss of job in a family member due to continuous travelling for the cancer treatment, loss of income in the patient due to their long-standing sickness, having to sell assets and liquidate all their savings to pay the hospital bills, ultimately leading them towards a very grim and uncertain future. The process of accessing cancer care was financially and emotionally challenging for many of the patients and their caregivers who were part of the study. It is interesting to note that despite this, most of those interviewed reported that they did not intend to compromise the prescribed treatment due to financial reasons.

***‘The family will not want that I suffer for my disease and hence they will try with all their heart and money to save me. But whoever has this disease they get finished financially’ (Patient #26, ovarian cancer patient).***

#### Applying for charitable funds from donors

The interviewing hospital being a not-for-profit setting, many patients interviewed were eligible for accessing charitable funds to cover treatment costs provided by the hospital as well as through other funding agencies. However, many patients were unaware of such help being available. The process for applying for donor funds was found to be challenging to some patients, given that they were already undergoing a complex treatment process.

***‘They gave me a list initially when tried to apply for funding. To organize all these documents was difficult …. So, it took a long time for me to apply. A lot of people don’t even know where to get an income certificate from. This process could be made a little easier for the patients’ (Patient #60, ovarian cancer patient)***

### Theme 6: Modifiers to accessing cancer care

#### Enablers of accessing cancer care

The interviews highlighted several enablers to accessing cancer care in India (Table 2). Bodily awareness, knowing others who had been treated for cancer, having trust in the medical system, support from family members, being involved in treatment-related decision-making helped patients to access care. Having the financial means to fund treatment was also an important enabler to access care.

#### Barriers to accessing cancer care

**Figure 2.**
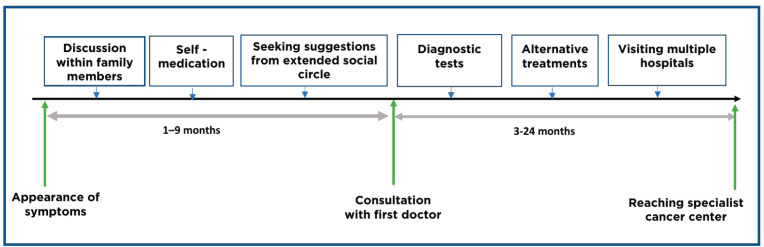
Pathways to the cancer centre.

On the other hand, lack of awareness, misinformation, lack of trust in medical establishments, having to travel far to access care, lack of involvement of the patient in treatment-related decision-making and financial hardships to support the direct and indirect costs of cancer care often contributed to the delays in getting treated for cancer. The enablers and barriers are described in Table 2. The patient interviews also reflected some of the time intervals of their cancer journey. The two intervals: a) from onset of symptoms to undergoing medical evaluations and b) onset of symptoms to reaching a specialist cancer centre are schematically represented in Figure 2. Following the initial symptoms, the patients often discussed their problems with their immediate family members and then within their extended social network. Some patients tried self-medication. In the present study only, 11% of patients accessed alternative forms of care as homoeopathy, Ayurveda and other indigenous systems of medicine. Our interviews elicited that the time interval between the onset of symptoms and first consultation ranged from 1 to 9 months. The next steps of diagnostic assessment and seeking multiple consultations in various hospitals often extended between 3 and 24 months before the patient finally reached a specialist cancer hospital for treatment. In this study, along with the qualitative information, a certain amount of quantitative data is derived from the interviews on the delay to reach a cancer centre and the reasons for the delay and this data has aided in reaching methodological triangulation [27].

## Discussion

This study highlighted six key themes that were related to a) the journey of patients to access care, b) the psychological journey of patients, c) stigma of cancer patients, d) decision-making and adherence to treatment, e) economic costs of cancer care and its impact and f) modifiers to accessing cancer care*.* The interviews highlighted that some cancer patients in India had travelled long distances and visited several hospitals before the diagnosis was confirmed and cancer-specific treatment could begin. The study found that the transition from primary and secondary care to the tertiary cancer centre was problematic for many of our participants. This has been reported to be an issue even in high-income countries with well-resourced health infrastructures [28]. Access to general health care in India is heavily influenced by the health-related inequity contributed by various socio-economic factors and cancer care is no exception [29]. Both genders were represented in the study sample while recruiting patients and their caregivers but men constituted 62% of the family members interviewed. This could be explained by the fact that in India patients often reside in extended families and have multiple care givers. Men prefer to accompany patients during their hospital visits while the home-based support may be provided by women.

The global cancer burden is predominantly in the LMICs. However, there is very little work from the patient’s perspective on social, cultural and economic determinants of cancer care and the impact of treatment experiences on patients from these parts of the world. A recent comprehensive meta-review of all the existing reviews since the 1950s on qualitative research done on adult cancer survivors reported that there is a paucity of published qualitative research beyond breast cancer, gynaecological cancers and prostate cancer survivors even from the high-income countries [30].

The transformation of the person into a patient as reported in this study is in line with the earlier work done on the nature of suffering experienced by an ill person [31]. The expression of this suffering is encompassed beyond the experience of their bodies. During the diagnosis and treatment of cancer, the patient may lose their personhood. The impact of the illness can challenge the sense of autonomy of an individual and reduce their willingness to participate in treatment-related discussions. The ensuing difficulties in treatment-related decision-making by the patient and his family members may result in treatment drop-out and non-adherence to treatment. Scholars have commented on this being possibly routed in fear and maybe a person’s way of coping with their predicament [32]. Work done in Sri Lanka on care pathways of women with breast cancer showed that patients take up numerous care paths following self-detection of a breast lump and several different specialists may be the first point of contact [33]. The above authors recommend empowering women to decide for themselves and expanding the network of dedicated breast clinics. A recent meta-analysis on stigma related to cancer has concluded that stigma impacts the emotional health of the cancer patient [34] and this was voiced by some of the participants of our study as well.

One limitation of the present study was that it was a single centre cross-sectional hospital-based study and as a result we could not include patients who never reached a cancer hospital. These patients constitute an important segment of cancer patients. Strengths of the present study are as follows: a) It was conducted in one of the largest cancer centres of the country and included participants from a wide geographical catchment area and the authors hope this would thus improve the representativeness of the sample. b) It had an inclusive design to encompass all common cancer types in India. c) The researchers used stratified purposive sampling [35] that captured major variations according to cancer types commonly reported from India as documented by the GLOBOCAN data for India [1]. d) The study had a robust sample size of 148 interviews and included a wide range of responders that included both patients and also their caregivers. e) The interviews were conducted by a clinical psychologist and a social worker both of whom had special experience of working with cancer patients. f) The research team had a variety of professionals who were not part of the oncology team who provided treatment to the patient. This helped the patients to be open and talk freely.

The diagnosis of cancer leads to an array of emotions in the patients as well as their family members. Other than getting treated, the treatment choices, participating in decision-making, having to face the socio-economic consequences of their treatment decisions are often overwhelming for the patients. In this study, insights on how they face and handle these issues throughout the trajectory of their illness have been explored. Other than few targeted works [36], there is not a lot of research done on the overall socio-cultural, economic and access-related experiences of cancer patients in India. This study is comprehensive in its approach in the way that it looked at the cumulative experiences as a whole and tried to understand how they impacted the patients’ journey through the disease and its treatment. It looked at the experiences on a broader landscape. The study has attempted to look at the patients’ experience with the disease in totality rather than in a fragmented manner. The interviews were analysed to understand what factors stood out to be most important to the patient in his or her journey with the disease rather than what factors are usually considered as important by the clinicians. The themes described are thus a combination of many factors ranging from psychological, to socio-cultural, economic, at systems levels as well as the physical experience of the disease itself.

We reported several barriers to accessing cancer care in India. The barriers may be within the social matrix and structural deficiency of health infrastructure near the patient’s home. Reports from Mexico have earlier identified very similar barriers to cancer care. This work discussed nine barriers to accessing cancer care that included financial burden, fear, problems posed by patient-provider communications, transportation and travelling long distances [37]. A report from Rwanda in Africa also highlighted financial, social and systemic factors leading to delay and disruption in cancer care in women with breast cancer [38]. Our finding of disruptions to health insurance coverage associated with financial hardships was also reported in high-income countries [39].

The need for comprehensive national health insurance scheme to support cancer patients has been highlighted for Nepal, a neighbouring country of India [40]. The Ayushman Bharat Pradhan Mantri Jan Arogya Yojana (AB-PMJAY) scheme has already been launched in India [41]. An initiative that is trying to address the fragmentation of cancer care across the country is the recently constituted National Cancer Grid (NCG) of India [42]. Earlier work had investigated disruptions to cancer care due to the Coronavirus disease-19 (COVID-19) pandemic [43] and mental health conditions [44]. This study highlighted the disruptions to cancer care caused by lack of comprehensive cancer care pathways, paucity of accessible cancer care services, lack of continuity of care between various service providers and other social factors as stigma.

## Implications for policymakers and possible solutions

We hope that our study will encourage health policymakers to keep in mind the experience voiced by the cancer patients while developing cancer services in India and beyond. Cancer patients need better and less fragmented care. It has been debated that a technology-centric approach to cancer care that invests only on high-end cancer care may do more harm than good [45]. We do agree to this and believe that equitable and more universal access to cancer care near the patient’s home will help patients. The powers of technology can be used in communicating between different cancer centres and creating health related database. The recent development of the NCG of India is surely a step in the right direction. NCG already has a network of 250 hospitals around the country. The aims of the NCG are to a) evolve and implement uniform standard of care across India, b) provide state-of-the-art cancer care to patients via one of its member hospitals, c) help train and create human resources needed to deliver cancer care, d) report on the epidemiological trends of cancer related data and e) provide leadership in conducting research on cost-effective cancer care [46]. Additionally, the AB-PMJAY scheme was approved by the government of India in 2018 to publicly fund health care of up to 500 million people living in India [41]. The present research has also identified the gap and disruptions in cancer care due to problems in decision-making and communication. We strongly believe that there needs to be better communication between patients and health care providers and culturally sensitive training in communication skills need to be developed and provided to clinicians.

### Implications for future research

Although not originally designed to be generalisable, the findings of this study resonate with studies done in other parts of the world with similar qualitative inquiry and the experiences of patients and their families are more similar than different, especially in the context of developing healthcare systems. Findings from this study would add value to further inquiries into the journeys of cancer patients, which may lead to identifying the deterrents of early and effective treatment of cancer. The enablers of optimum care delivery may also be explored further in different parts of the world.

## Conclusion

The transition of cancer patients from primary and secondary care to oncology hospitals may be fraught with several logistical difficulties. At a psychological level, the patients and families go through an emotional journey as well. The financial impact of cancer care is a serious issue for many patients who must fund their treatment with help from friends and family members. The paper highlights this journey in India and some of the findings may apply to several LMICs with similar health infrastructure. Future research is needed on service delivery models for cancer care suited to the LMICs. Health policymakers will need to consider the impact of the cancer journey on patients and develop care pathways that are inclusive and address some of the barriers identified in this study.

## Conflicts of interest

The author(s) declare that they have no conflicts of interest.

## Funding statement

This project was funded by The Wellcome Trust, UK.

## Figures and Tables

**Table 1.** Respondent characteristics.

Variable (*n* = 148)		Patients	Caregivers	
	*n* = 100	Percentage	*n* = 48	Percentage
Gender				
Male	35	35%	30	62.5%
Female	65	65%	18	37.5%
Age range (years)				
18–29	1	1%	6	12.5%
30–39	0	0%	14	29.2%
40–49	20	20%	13	27%
50–59	20	20%	9	18.75%
60–69	35	35%	5	10.42%
70–79	21	21%	1	2.1%
80–89	3	3%	0	0%
Education level attained				
No formal education	23	23%	0	0%
Primary	11	11%	7	14.6%
Secondary	18	18%	9	18.75%
Graduation	20	20%	24	50%
Technical/others	25	25%	2	4.16%
Post-graduation	3	3%	6	12.5%
Cancer			Cancer (index patient)	
Breast cancer	25	25%	11	22.9%
Gynaecological cancers	21	21%	4	8.3%
Head and neck cancers	11	11%	13	27.1%
Gastrointestinal cancers	22	22%	10	20.8%
Haematolymphoid cancers	6	6%	5	10.4%
Genitourinary cancers	9	9%	1	2.1%
Lung cancer	6	6%	4	8.3%
Relation of primary caregiver with cancer patient				
Husband			5	10.4%
Wife			7	14.6%
Son			14	29.2%
Daughter			7	14.6%
Father			1	2.1%
Son-in-law			2	4.16%
Daughter-in-law			1	2.1%
Brother			5	10.4%
Sister			2	4.16%
Cousin			4	8.3%

**Table 2.** Barriers and enablers for help-seeking in cancer.

Enablers	Quote	Barriers (Delay inducers)	Quote
Knowledge on the symptoms and bodily awareness	‘I was alert. After 35 I used to do self-examinations. But many people do not do this. This is not only for breast cancer but for any other type of cancer. Sometimes there is weight loss, and we may think that we have not been eating properly or something similar. This is how it gets late to diagnose. I identified early as I was alert’ (ID # 7). t.	Lack of awareness	‘We didn’t think of cancer. We were scared of endoscopy. It took us a lot of time. Maybe if we did the endoscopy quickly, it would have been detected earlier’ (ID # 21).
Knowing someone who had undergone treatment for cancer	‘One of my friend’s mother has got treated here. He told me about this hospital. So, he (my friend) helped a lot’ (ID # 38).	Misinformed by people with similar symptoms who did not have cancer	‘I never had any idea that the cysts could give rise to other illnesses in future. Everyone advised me that most women take homeopathic medicines for cysts and that cures them’ (ID # 2).
Trust on treatment centre and doctor	‘To be very honest, because of trust I came here. XX is a specialist cancer hospital, everybody comes here If they have cancer’ (ID # 93).	Distrust in local doctors and perceiving that wrong treatment given locally	‘No, actually we have undergone treatment at many places. Initially, no one could detect it. After the MRI they should have known that there is something wrong. Here all the tests were completed within no time’ (ID # 46). ‘We took him to XX but couldn't trust that hospital’ (ID # 108).
Word of mouth from trustworthy local people about a faraway hospital may facilitate travelling for treatment	‘The doctor who saw her first said that she got operated on from here for her cancer. She recommended this place’ (ID # 40). ‘No, we have not faced any problem. We have met with other patients from Bangladesh’ (ID # 91).	Travelling far away to get treated for cancer can also be a barrier to treatment adherence due to the disruption that it poses on life in general	‘We don’t have any good hospital near our place. They can’t even diagnose properly. Cancer treatment is available only in the cities. It is difficult for villagers to know much about the cities. People are busy and they don’t come to the city often. Even when I come, I apply for leave from my employer. So that is why maybe people don’t know about these hospitals and die without any treatment’ (ID # 48). ‘It's been 25 days since we are here. We are looking forward to going back to Tripura as soon as possible. There are monetary constraints as well. My son has his exams and that's my priority. It's because of my illness; my son is missing out on his exams. It's all because of me that he is not getting a job. Moreover, it's been a long distance away from home and I am not feeling good. If I come back for treatment, then I must stay at Tripura Bhawan. The food at Tripura Bhawan does not agree with my palate. But I must eat it. I don't feel like staying there. I have a doctor's appointment tomorrow. If he suggests that further treatment can be done at Tripura then I shall go back home’ (ID # 1).
Support of family, extended family, work colleagues and friends	‘But immediately after my symptom started, I spoke to my daughter, and I spoke to my family. We came here for my treatment. I am very careful about my health’ (ID # 59).	Lack of support from family and friends may be a barrier in accessing timely help and making the right decisions	‘My daughters are married; they have their own family to take care of. I don't get help anybody other than my wife’ (ID # 98). ‘Who will take care of my kids? They don't have any uncles; they only have an aunt. So, I am struggling to survive’ (ID # 42).
Financial support Health insurance Support from family	‘Luckily, I have health insurance. With my insurance and savings, I think it will be done. Financially it’s not too stressful for me, I think I will manage’ (ID # 7). manage. ‘There aren't any financial strains. My siblings love me a lot. They want my speedy recovery. They will support me financially no matter what. Since my parents’ demise, my sisters have been living with me. I am their only family. They have sold all their land for me’ (ID # 1).	Financial problems No Health insurance. Health insurance that could not be renewed. Being supported by family and friends financially. Running out of funds and choosing a less expensive treatment option.	‘I didn’t have any insurance. That was my biggest mistake. I think I should get one now. I might face problems again in future’ (Patient 60). ‘I had a Mediclaim (insurance) but it lapsed due to a misunderstanding. I couldn’t renew it’ (ID # 67). ‘I had spent fifty-sixty thousand rupees from my pocket. My uncle gave me two lac rupees. The rest of the money I had to borrow from people. I have four children and my husband is undergoing dialysis. He is also seriously ill. Even the hospital supported me by funding fifty thousand rupees of my treatment’ (ID # 42). ‘I have sold it all. My wife doesn't have a single piece of gold jewelry left to sell. We have sold everything. Now I don’t even have any assets left to sell. I am now consulting a homeopathy doctor in Calcutta as it doesn’t have any side effects and it's very cheap. I don't have money for the expensive treatments. How much money will the others give me? They have their own families’ (ID # 100).
Decision-making by the patient facilitated quicker treatment	‘She can think for herself. Till now she used to do everything by herself. How much money to invest and where? Everything was managed by her. My mother is very aware of her disease. Now she is realizing that we are spending a lot of money on medicine and tests. The day before yesterday, my mother told me that we must go to the hospital again’ (ID # 71). ‘The ultimate decision had to be made by me because I am the primary decision-maker. Lots of people also told me to take her to Mumbai but being on my own I couldn't take her to Mumbai’ (ID # 87). ‘I had to make all the decisions. I am the patient. My father is old. And my mom is old too. People who assisted me didn’t give me any final decision. They said you must take the final call. We will accompany you. They were scared that if something went wrong, then they will be held responsible. So, I thought if I had to take a call, I will also think financially’ (ID # 38).	Lack of information and indecisiveness led to delays	‘So far we are unable to make any decision. It’s very difficult. A lot of people have deteriorated after taking chemotherapy. That’s why we are very scared. I have also spoken to doctors and came to know that no one gives a guarantee that after taking chemotherapy people will lead a healthy life for two to three years. That might not happen. That's why I am feeling helpless and emotionally disturbed. I would say that scientists have reached a stage where only people who are detected with cancer in the early stages have an answer. But that’s not a lot of people’ (ID # 85). “I was suggested a few tests in XX. I couldn't make the decision. So, they discharged me from there and we went to XX (teaching hospital located far away from home). There they did a lot of tests and then they said you don’t have anything in your heart. You should consult a gastroenterologist. So, we went to a gastroenterologist in XXXX. He did a lot of tests and he said you have a tumor and an ulcer in your food tract. He showed me all the scan pictures. Then from there, I went to Chennai to a cancer hospital and a surgeon reviewed me and said that ‘you have problems related to food pipe and if you want to get treated you have to stay here for the six months. He added that as I have language barriers, it's better that you go to XX hospital, that is in your hometown, and referred me to here’” (ID # 99).
